# Composition and Therapeutical Uses of True James’ Powder

**Published:** 1879-08

**Authors:** P. O’Connell

**Affiliations:** Chicago, Ills


### ARTICLE V.

### COMPOSITION AND THERAPEUTICAL USES OF TRUE JAMES' POWDER. By P. O'CONNELL, M.D.

Reference is here had only to the genuine James' powder (*Pulvis Jacobi Verus*), and not to the common substitute, if not worthless compound, which usually goes by that name; I mean the *pulvis antimonialis* or compound powder of antimony of the pharmacopœias.

A great deal of discrepancy exists among the highest authorities with regard to the composition and mode of preparing James' powder. I regret that many attempts on my part to arrive at a correct knowledge of the chemistry of this valuable medicine have not, so far, been as successful as I would wish. Standard works on therapeutics and pharmacy say but little about it. Private correspondence with distinguished chemists and pharmaceutists has not been very successful in removing the veil of secrecy which seems to cling round this article. Beyond saying that it is an antimonial, I know but little that is certain with regard to its composition.

Dr. James, who died in 1776, and after whom the article is named, was the first to prepare this nostrum. The American Dispensatory, edition of 1871, says: "It is prepared by calcining, in an iron crucible, one part of tersulphuret of antimony and two parts of horn shavings, stirring constantly until vapor ceases to rise. Then rub the residue to powder, put it in a crucible with a perforated cover and raise it gradually to red heat, which must be maintained for two hours. Reduce the product, when cold, to fine powder. It forms a white, gritty, odorless, tasteless powder, partially soluble in boiling water, and which solution gives an orange-yellow precipitate with sulphuretted hydrogen. It possesses the general properties of the antimonials, according to the doses in which it is employed, and like them is very uncertain in its operation. It is principally used as a sedative and diaphoretic in febrile diseases, in from one to four grain doses, in powder, every three or four hours." The genuine article is not so definitely known as this; while, so far as my experience of its use has gone (and it has not been small) I find it most reliable in its action. Its tastelessness, too, is a very strong recommendation in its favor, since it can be administered to children however young, when quinine, owing to its bitterness, is obstinately and absolutely refused.

The Ameriean reprint of Cooley's Cyclopedia of Practical Receipts, fifth edition, edited by Richard V. Tuson, F.C.S., London, says: "The antimonial powder, or compound powder of antimony of the pharmacopœias, is the preparation which usually passes under this name; but the true James' Powder is a nostrum the pretended secret of the preparation of which is claimed to be possessed by only two parties in the Kingdom. The patent specification of the once celebrated Dr. James runs as follows: 'Take of antimony, calcine it with a continued protracted heat, in a flat, unglazed earthen vessel, adding from time to time a sufficient quantity of any animal oil and salt well dephlegmated; then boil it in melted nitre for a considerable time, and separate the powder from the nitre by dissolving it in water.' It has been remarked that this yields a product totally different from that which Dr. James and his successors have sold under the name. Hence he has been charged with concealing the true formula for his powder, and of publishing a false one in its stead."

According to Dr. Robinson, the original formula for this nostrum, and that still adopted by the vendors of the proprietary article at the present day, is: Tartarized antimony 1 part; preprepared burnt hartshorn and calx of antimony, of each 5 parts; carefully mixed together and divided into 21 grain powders. (*Phil. Journ. Pharm.*, vi, 282). Never having witnessed the slightest depression nor nausea, in young or old subjects, follow its use, I do not think the preparation contains any tartar emetic. "Antimonial powder differs from true James' Powder, in which I have found a protoxide of antimony, to which it owes its efficacy, whilst the antimonial powder contains antimonious acid which is inert."—Thompson's London Dispensatory.

Mr. A. W. Gerrard, F.C.S., Teacher of Pharmacy, University College, London, informs me by letter under date Nov. 7, 1878, that James' powder "consists essentially of a mixture of phosphate of lime, antimonious acid, and oxide of antimony."

"From analyses recently made of these specimens of James' powder (Newberry's, Butler's, and a sample 60 years old obtained by Mr. Squire), it appears that antimonious acid was present in varying proportions, from about 45 per cent. to 33 per cent., the amount being greatest in the old specimen; teroxide of antimony was also present to the extent of from 9 per cent. to 1 per cent., the greatest quantity being again in the old preparation; the remainder in each specimen consisted chiefly of phosphate of lime; no trace of tartaric acid was discoverable in any of the specimens."

No two authorities, we see, are agreed as to the composition of this medicine. It contains, according to most writers, protoxide of antimony and antimonious acid. The analyses are, doubtless, correct and therefore to be relied on. The various ingredients, then, found in true James' Powder, with their action and doses, may be summarized thus:

1. Oxide, protoxide, or teroxide of antimony, which is a grayish-white powder, is used chiefly in preparing tartar emetic and in making *pulvis antimonialis*, of which it constitutes one part in three. It is diaphoretic and febrifuge. The dose is from one to ten grains. It is very unreliable, being sometimes very active and sometimes inert, owing doubtless to differences in the manner of its preparation. It is said to be very mild when well prepared, and deserves, perhaps, to be more used. It is obsolescent.

2. Antimonious acid (synon. antimonic tetroxide). Some believe it is inert; this may fairly be doubted. It constituted about one-half to one-third of the three specimens analyzed by Mr. Squire. To it chiefly, probably, is due the remarkable efficacy of the drug.

3. Calx of antimony. This is a white or grayish-white powder, tasteless and inodorous. It is greatly diaphoretic and laxative. The dose is from one to ten grains. It is not now employed.

4. Phosphate of lime. It is used by some on the supposition that it increases the growth of bone. Believing that it hastens and stimulates the growth of cellus, the French use it largely to promote the union of fractures. Dose from twenty to sixty grains, three times a day.

To what particular ingredient or ingredients this preparation owes its efficacy, I cannot positively say; but I do know that, in my hands, it has proved a most useful as well as reliable medicine. "James' powder is used in both acute and intermittent fevers, and in all febrile and inflammatory diseases. It is more mild and more uniform in action than the antimonial powder of the pharmacopœia. Perhaps no nostrum ever received such extensive patronage from the faculty as James' powder. Dr. James himself was remarkably successful in its use; but whether his success depended upon his powder or on the mercurials and bark which he commonly employed at the same time, is still undetermined."

With the view of determining, if possible, this point, I made comparative trials of James' powder alone, and with the drug in combination with quinine, calomel, and sometimes with opium. Of twenty-two cases of grave sickness, of which I preserved notes, in which the James' powder alone, or combined with the above named drugs, was used, the following is a very brief summary:

Four cases were intermittent quotidian fever in young children; two were puerperal pyrexia; one was pyrexia, following profuse—almost fatal—post-partum hæmorrhage, on the eleventh day after delivery; three were puerperal peritonitis (two acute and one sub-acute of three weeks duration); two were acute puerperal metritis; one was acute peritonitis in a woman the subject of menorrhagia; one was acute metritis and pelvic cellulitis, with effusion; and eight were cases of acute pneumonia, all lobar, some double and some of the entire lung. All made rapid and complete recoveries, with the single exception of one of the cases of acute puerperal peritonitis, when the patient after the subsidence of the peritonitis sank from exhaustion due to pelvic effusion.

*In two*, of the twenty-two cases, James' powder, calomel and opium combined were used; *in one case*, James' powder, quinine and opium combined were used; *in three cases*, James' powder and quinine combined were employed; *in three cases*, James' powder and opium combined were used; and *in thirteen cases*, the James' powder alone was administered. The thirteen cases in which the James' powder alone was employed, included two cases of intermittent fever, one of puerperal pyrexia, the case of pyrexia following the post-partum hæmorrhage, one of acute metritis and pelvic cellulitis with effusion, one of puerperal metritis, and seven cases of pneumonitis. In these thirteen cases the action of the James' powder alone was just as rapid, and the effect as complete and as satisfactory, as when combined with the quinine, calomel or opium.

The temperature was observed in all the cases with the exception of two of the children suffering from the intermittent fever, in whom, owing to fear and restlessness, I could not note the temperature accurately. The twenty cases gave an average temperature of 39.2°. Their average temperature, after an average use of the drug, every four hours, for three days, was 37.2°. The average temperature of the seven cases in which the James' powder was used *in combination* with quinine, calomel and opium, was 39.1°; and the average temperature of the thirteen cases in which James' powder *alone* was prescribed, was 39.2°. The average temperature, after an average use of the drugs for three days, was, in the seven cases, 37.1°, and in the thirteen cases, 37.2°. The average duration of illness—that is, to the beginning of convalescence—was seven and three-fifths days in the whole twenty-two cases. In the thirteen cases in which James' powder alone was employed, the average duration of the disease was six and eleven-thirteenths days; in the other nine cases, the average duration of illness was seven days.

These facts help to determine that it is *not to the mercurials and bark*, but to the *James' powder alone*, the speedy reduction of the temperature, with complete as well as rapid resolution of inflammatory and febrile diseases, is due.

Quinine is now extensively employed as an antipyretic; but to make a decided impression on the temperature, one to two grams, repeated once or twice in twenty-four hours, are necessary. A few such large doses are certain to induce the disagreeable effects of the drug on the brain, and the patient will complain bitterly of the tinnitus aurium and of his vision. Its very bitterness, too, constitutes no small difficulty and drawback in the administration of this invaluable medicine, which, in the case of children, is well-nigh insuperable. The writer, even, cannot take quinine, owing to the nausea and vomiting which its bitterness induces. With James' powder no such difficulties are encountered, and no disagreeable effects follow its use; while such small doses as 20 to 30 Ctg. to adults, every two, three, four or six hours, as may be required, need not be exceeded, and will produce, but more slowly, the same depression of temperature as 1 to 2 grams of quinine once or twice a day. Being tasteless, it can be taken with or without sugar, by young or old, on the most delicate stomach. Its cost, too, is only about half that of quinine at present rates.

It is diaphoretic, antiperiodic and antipyretic. Its diaphoretic action is gentle and moderate. As an antiperiodic, in the intermittents of children, I find it quite as prompt and as effectual as quinine. As an antipyretic, I notice a steady decrease in the temperature and in the pulse rate as soon as its administration is commenced. It does not, any more than quinine, prevent an increase of temperature when a fresh focus of inflammation is lighted up, such as the implication of a fresh lobe of the lung during the progress of a case of pneumonia. It seems to exercise a specific effect in puerperal inflammations in particular, arresting the morbid action, whether it be a metritis, cellulitis or peritonitis, causing a very speedy and complete *resolution* of the inflammation, with return of the lochia. It appears to exert a very marked beneficial action in pneumonitis also.

Chicago, Ills , July 5, 1879.

---

Chlorhydrate of Pilocarpine in the Production of Premature Labor. Dr. Chantreuil. (*La France Médical*, Nos. 28, 30, 33, 34, 1879.

Dr. Chantreuil, at the *Hôpital des Cliniques*, after advocating at length the claims of hydrate of chloral in the treatment of eclampsia and an absolute milk diet as a prophylactic of the same when albuminuria is present, discusses the reliability of pilocarpine in the production of premature labor. Before narrating his own experience, he calls attention to the observations of Massmann, who had twice seen labor produced by the hypodermic injection of pilocarpine, and to the experience of Schauta (assisted by Spaeth) and Kleinwachter, both of whom had witnessed a case of labor brought about by the same agent. The doses employed by these gentlemen varied from 0.07 to 0.053 of the chlorhydrate of pilocarpine. To confront these apparent successes, he adduces the experience of others—in all eight cases—in which much greater doses failed entirely. After narrating the experience of M. Hyervaux and himself on rabbits, dogs and guinea-pigs, and claiming that the success of those who had observed positive results could be explained by other exciting causes, he concludes that it is unwise to rely on pilocarpine to produce premature labor in a case of albuminuria. While admitting that it may assist labor after the dilatation of the os, he himself would exhibit it only with great reluctance on account of its cardiac action.

